# Supplementary figures and images for: Contrast enhanced longitudinal changes observed in an experimental bleomycin-induced lung fibrosis rat model by radial DCE-MRI at 9.4T
Source: PLoS One. 2024 Sep 27;19(9):e0310643. doi: 10.1371/journal.pone.0310643 (PMC11432896; doi:10.1371/journal.pone.0310643)

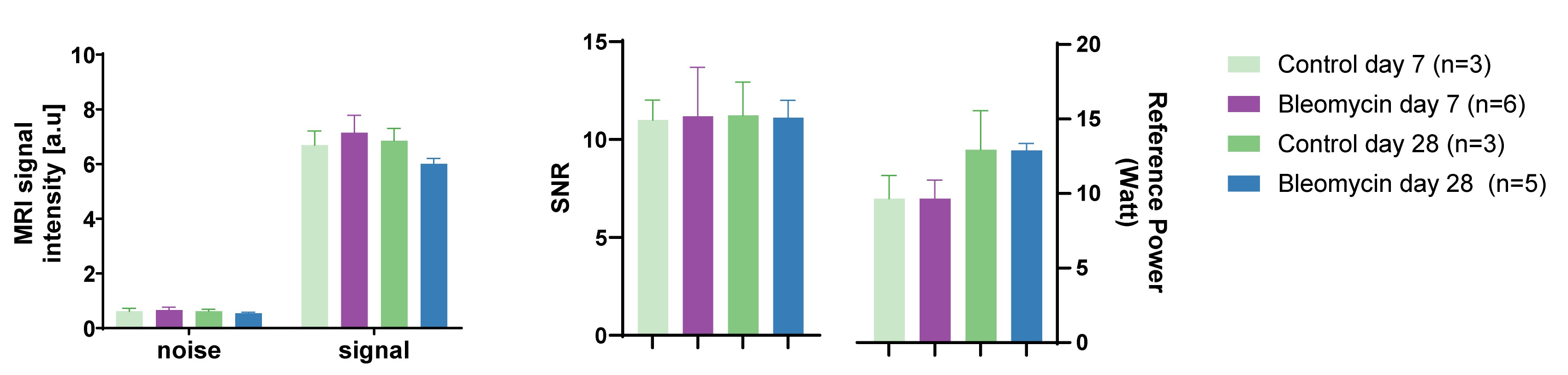

Supplement: S1 Fig — Increase in animal weight will change the load of the RF coil and potentially leads to changes in image quality. To establish the signal to noise over time, the noise level and the signal level were measured in an image used for localization purposes. Here, conventional Cartesian scanning was employed, after which the signal to noise ratio was calculated. Further, the power levels needed for the scanning established by the MRI scanner were plotted as well. Clearly, more power was needed for the animals on day 28, but the signal to noise ratio of the images was not different between the animal groups and time points. (TIF) [file pone.0310643.s001.tif]

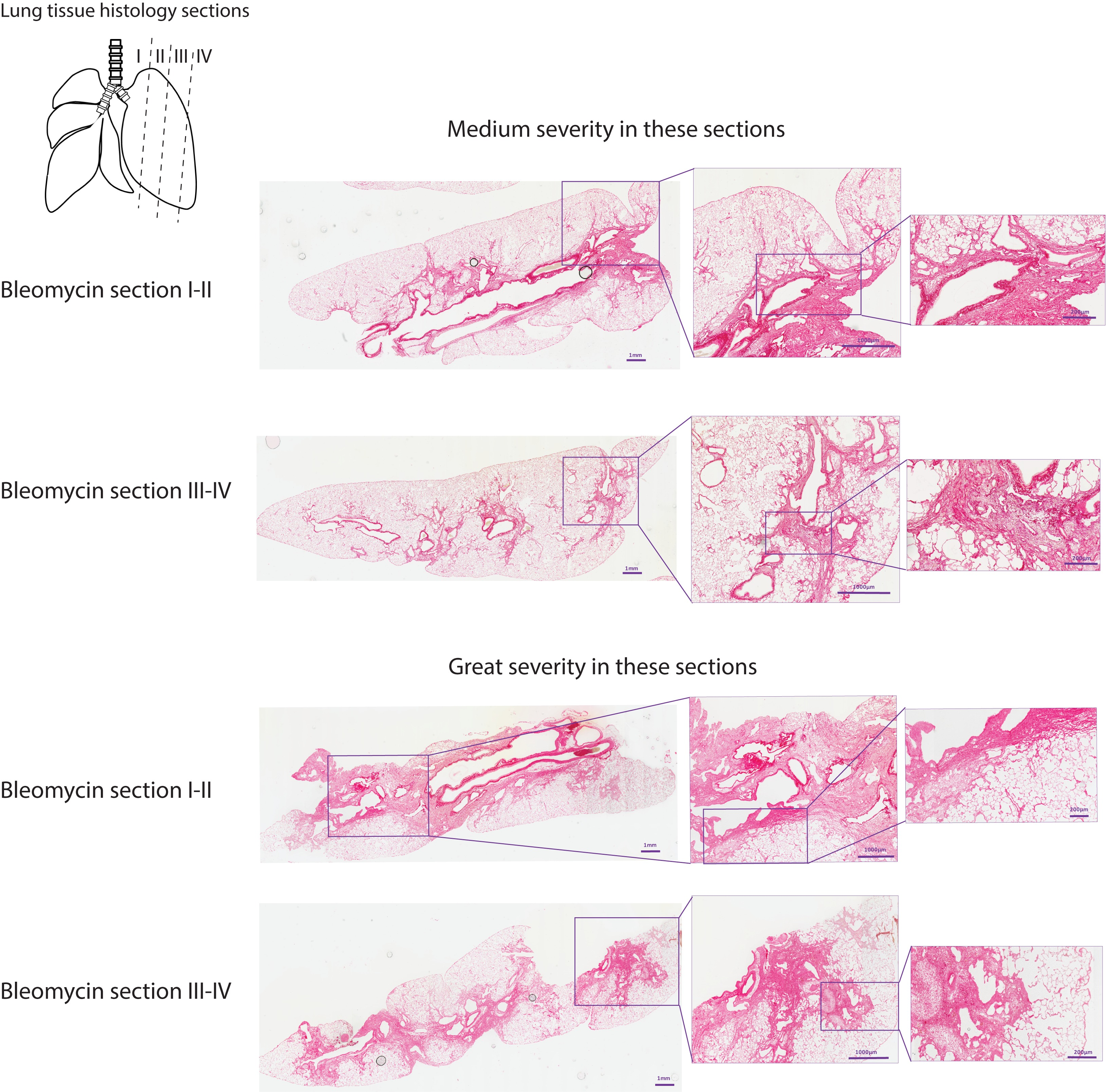

Supplement: S2 Fig — Lung tissue sections were stained using Picro Sirus Red staining kit (Collagen: Red, Muscle Fibers: Yellow, Cytoplasm: Yellow). Clearly, the presence of collagen can be visualized, and heterogeneity is depicted for different disease severity and locations in the lung. (TIF) [file pone.0310643.s002.tif]
