# Supplementary material for: Contrast enhanced longitudinal changes observed in an experimental bleomycin-induced lung fibrosis rat model by radial DCE-MRI at 9.4T
Source: PLoS One. 2024 Sep 27;19(9):e0310643. doi: 10.1371/journal.pone.0310643 (PMC11432896; doi:10.1371/journal.pone.0310643)
Supplement: Attachment — Submitted filename: Response to reviewers.docx [file pone.0310643.s003.docx]

## Comments to Academic Editor

1. Please ensure that your manuscript meets PLOS ONE's style requirements, including those for file naming. The PLOS ONE style templates can be found at
   <https://journals.plos.org/plosone/s/file?id=wjVg/PLOSOne_formatting_sample_main_body.pdf> and
   <https://journals.plos.org/plosone/s/file?id=ba62/PLOSOne_formatting_sample_title_authors_affiliations.pdf>

*We have double checked the style requirements and all requirements are met as far as we can judge.*

*Changes implemented:*

*- short title removed from title page and only mentioned in the submission system.*

*- reference system Vancouver style adapted from (x) to [x] according to your guidelines*

*- changed the level sections in the material and method section from level 3 to level 2.*

*- all figures have been processed through PACE without any issues. The PDF files were visually inspected and the processed TIFF files from the PACE page were used for this revised submission.*

2. Please note that PLOS ONE has specific guidelines on code sharing for submissions in which author-generated code underpins the findings in the manuscript. In these cases, all author-generated code must be made available without restrictions upon publication of the work. Please review our guidelines at <https://journals.plos.org/plosone/s/materials-and-software-sharing#loc-sharing-code> and ensure that your code is shared in a way that follows best practice and facilitates reproducibility and reuse.

*All material needed to reproduce the findings will be available at the Swedish National data service. A DOI will be activated upon accepting of the manuscript, and the following link will be available for review and this repository will become public on acceptance of the manuscript.*

*https://doi.org/10.5878/b4cg-vc79 (not activated yet)*

*During the review process, the repository can be viewed using the following URL.*

*https://snd.se/en/catalogue/dataset/preview/8078bcbf-8fc9-4c71-9119-eb4682d7542f/1*

3. Thank you for stating the following financial disclosure: "TRISTAN-IHI consortium (#IB4SD-116106, <https://www.imi-tristan.eu/>) (Translational Imaging in Drug Safety Assessment - Innovative Health Initiative) "
Please state what role the funders took in the study. If the funders had no role, please state: ""The funders had no role in study design, data collection and analysis, decision to publish, or preparation of the manuscript."" If this statement is not correct you must amend it as needed.
Please include this amended Role of Funder statement in your cover letter; we will change the online submission form on your behalf.

*We would like to use the following statement, and this is also mentioned in the cover letter.*

*The research leading to these results received funding from the Innovative Medicines Initiatives 2 Joint Undertaking under grant agreement No 116106 (IB4SD-TRISTAN). This Joint Undertaking receives support from the European Union's Horizon 2020 research and innovation programme and EFPIA. The funders had no role in study design, data collection and analysis, decision to publish, or preparation of the manuscript*

## Reviewers' comments: Reviewer #1

In this manuscript, René in ’t Zandt et al describe the application of DCE-MRI to a rat model of bleomycin-induced lung injury. The experiments and analyses were performed well and carefully. The work is novel, as far as I’m aware, no publication exists yet concerning DCE-MRI applied to a bleomycin model.

Major comments

-Animals: 15 rats were used in the study (line 99). In figure 2, data from 6 rats are shown as controls. Therefore, I presume that 9 rats received bleomycin. However, in figure 2, data from 6 rats were summarized at day 7 and data from 5 rats were summarized at day 28. I suppose that the same animals were measured at both time points after bleomycin. Thus, why data from only 6 respectively 5 rats were summarized instead of 9? More clarity about the experimental procedure should be provided regarding this point.

*The following was added to the material and methods to clarify the animal group sizes.*

*A total of 15 animals were included in this study. 4 animals were used as controls (receiving only Saline instillation) and 11 animals received bleomycin. Based on prior experiences with the bleomycin model, 5 out of 11 animals were designated as backup subjects from the imaging group of bleomycin exposed animals, to maintain the study's reliability and continuity in case of unexpected issues with the primary subjects, such as extensive weight loss or imaging or anesthesia related events. Day 7 and day 28 were chosen for DCE-MRI scan days as two representative days of dominant inflammatory and fibrotic time points respectively. The planned group size for DCE-MRI in bleomycin exposed animals was n=6.*

*We also added the following section to the Results section.*

*DCE-MRI was successful in 3 out of 4 control animals on day 7, for 1 animal the Venflon catheter punctured the venous artery and substantial amount of fluid was injected extra-vascular in the tail. This animal was removed from the DCE-MRI on day 28 due to vascular damage in the tail. Subsequently, only 3 controls were available for DCE-MRI on day 28 as well. In the bleomycin-exposed group, all DCE-MRI injections (n=6) were completed successfully on day 7. On day 28, one contrast agent injection partly failed as the images showed that only a fraction of the contrast agent reached the lung. This reduced the group size for DCE-MRI in bleomycin-exposed group animals to n=5.*

-Figure 2: Enhancement factor is by definition a factor and does not have units. Therefore, a.u. (arbitrary units) should be omitted. Also, the quality of the graphs in the pdf document was poor – increasing the font size and the quality of the graphs reproduction would be welcome.

*Thank you for this comment. The authors agree on this point and have removed this label from the figures when enhancement factor was plotted on the y-axis.*

*We agree that the quality of the graphs in the pdf document provided for review is poor. The link provided on the right top corner on the appropriate page in the submission pdf file we have, contains links to the tiff version of the files. These files have the quality required by PLOS ONE as far as we can judge. For the resubmission, we have used the PACE diagnostic tool as recommend by PLOS ONE to make sure the quality requirements are met in this revision.*

-The colors are confusing. In fig. 2a the rectangles around the images are green, blue and purple for control, bleomycin day 7 and bleomycin day 28, respectively. However, in fig. 2b, the colors are green, purple and blue for control, bleomycin day 7 and bleomycin day 28, respectively. Please adapt the colors. In order to better discriminate control curves, I suggest plotting them with black fonts.

*Thank you for this notification. Unfortunately, the rectangular color boxes surrounding the images in figures 2 and 3 were switched for animals measured on day 7 and day 28. We have fixed this and apologize for this confusion.*

-Lines 272-273: The fact that the tissue remodeling detected by MRI occurs primarily around main airways has been shown earlier and confirmed histologically by comparison observations in the same regions (doi 10.1002/jmri.22476).

*This is a good point; we have mentioned this in the manuscript and included this reference*

-Signal intensity of the tissue remodeling area around main airways, determined from images before the contrast agent injection, should also be provided. Analysis should be compared with the DCE-MRI results. It might be the case that both measures might be able to discriminate between the inflammatory and more fibrotic (tissue remodeling) phases of the bleomycin model.

*Thank you for this comment and we realize that adding the tissue remodeling/lesion area would improve our work. We lack the same gradient echo sequence used in previous publications to determine these areas in the lung. Therefore, we reconstructed a new baseline image that uses the data acquired over 5m40s prior to the injection of the contrast agent. The histogram analyses were performed like what has been described previously by our group. The volume of the pixels with an intensity higher than the intersection with the x-axis are calculated, plotted and added as figure 1C in the manuscript. The pixels showing the area of this ‘lesion/tissue remodeling’ have been added to figure 3* *as an extra overlay to the MRI images.*

*We have not been able to reveal any correlation between the tissue remodeling and lesions area neither have we been able to see any changes in the washout rate for the binned DCE curves.*

-Figure 2B,C: It is not clear to me whether the curves represent the mean of an average of signal over the whole lung in every animal, or whether they are the mean of the signal over the lesions in every rat. I rather suppose that it is the first, as the mean profiles look the same in control and bleomycin animals (except obviously for the peak), which is surprising to me. I would expect the washout period in bleomycin rats to be different (longer) than in controls. I would strongly suggest to perform analyses in the areas around main airways as well, where most of the remodeling occurred, and present the results in a separate figure. Please clarify.

*The curves shown in figure 2B represent the average pixel intensity curves over the total lung. We have added ‘total’ to the text to emphasize that.*

*The washout period being longer in bleomycin rats was also one of our assumptions to prove. We can only conclude that based on our data we cannot show that. As explained above, the binning approach didn’t reveal any difference in the dynamics. But these are the dynamics for pixels without any connection to its anatomical location in the lung.*

*Looking at the volume that each bin class represents in the lung, a clear difference can be observed for the bleomycin-exposed animals. Interestingly, this difference was found for the lower signal intensity pixels rather than the high intensity pixels who correspond to the lesions determined by the histogram analysis. Pixels with lower signal intensity are mostly found on the lateral side of the lung lobes and, although it would be tempting to attribute this to collagen formation, the location doesn’t match the presumptive dominant lesion area and it is more likely that “simple expanded lung volume” with reduced tissue density is the main explanation for the finding observed. This is also what we argue for in our manuscript.*

*Analysis close to the major airways is a challenge for the DCE experiment due to the high signal intensity of the vessels and the need to analyze the dynamics in tissue in proximity with these vessels and airways. The vessels pulsate with the heartbeat and the respiration creates substantial movement of tissue. The data shown is not corrected for either of these physiological processes and signal bleed is to be expected to play a major role*

-Figure 3: The same comment concerning color coding made above for figure 2 holds true here.

*The authors agree and the error has been fixed*

-Figure 3B: If I understood correctly the distribution of the clusters and compare to the images shown in figure 3A, does it mean that the higher cluster volumes for lower enhancement factors at day 28 after bleomycin are related to diffuse fibrosis? This would be extremely interesting – especially when testing a therapy and comparing the results to the stronger remodeling around main airways in the central lung. Please clarify.

*This would be the preferred conclusion, but we have no real proof of that. It is more likely this is correlated with the increased lung volume, a decrease in cell density and therefore an apparent lower MRI signal. Histology supports this observation. We opted to go for the most likely explanation based on the lung volume differences found.*

-Discussion: In the Introduction section, the authors mention references 8-10 summarizing clinical DCE-MRI work in IPF patients. They should briefly compare their findings with the clinical observations.

*We agree and have modified a section in the discussion to reconnect to that section in the introduction, with the modification underlined.*

*….Substantial inflow effects and the relative low time resolution compared to the mean transit time in the rat lung [11], hampers a model-based analysis in our experiments. Additionally, these factors complicate the direct translation of perfusion-sensitive DCE-MRI used clinically, as this application relies on the measurement of the first-passage characteristics of the injected contrast agent [9, 10]. It cannot be excluded that using a scanning DCE approach with significantly higher temporal and spatial resolution more details in the bleomycin model could be revealed…..*

-Contrast agent: Given the fact that the use of Gd-based contrast agents in MRI examinations is being scrutinized due to reported side effects, especially in the kidney, the pros/cons of DCE-MRI in the evaluation of pulmonary fibrosis in comparison to MRI techniques not relying on the administration of contrast material should be briefly discussed.

*Patients with idiopathic pulmonary fibrosis are known to have a higher prevalence of chronic kidney disease, which is also regarded as a comorbidity. The prevalence of chronic kidney disease in this patient group increases with age, mirroring the age-related increase seen in patients with idiopathic pulmonary fibrosis itself. In a clinical setting, patients referred to an MRI-examination are checked for potential contraindications, e.g. contraindications to receiving gadolinium-based contrast agents, which potentially can cause nephrogenic systemic fibrosis. For patients with known or suspected kidney disease a blood test is performed to measure the level of creatinine and to calculate the estimated glomerular filtration rate. Depending on the result the patient may or may not undergo the MRI-examination with a gadolinium-based contrast agent.*

*The above discussion is well-known and of course important for clinical application of DCE-MRI to IPF-patients. Our paper focuses on the translational capability of DCE-MRI to a preclinical setting, and we argue that the above discussion is beyond our scope.*

Minor comments

-Line 74: Suggest replacing “In addition” by “Also”.

*We have adapted this.*

-Line 86: Suggest replacing “Therefore” by “Thus”.

*We have adapted this.*

-Lines 152-153 & lines 167-221: Sentences in past tense would be more appropriate

*We have adapted this.*

-Results should be described in past tense (e.g. line 257).

*Thank you for pointing out this inconsistency, we have checked that all is written in the past tense throughout the results section and further checked the manuscript on tense inconsistencies*

## Reviewers' comments: Reviewer #2

In the manuscript the authors present their work on dynamic contrast enhanced lung MRI in a rodent model in which lung fibrosis was induced using bleomycin.
For this n = 15 rats were used, of those one group and I went a bleomycin challenge at day 0, the second group served as control and did not underwent any treatment. Lung MRI was performed at day 7 and day 28 using using a 9.4 Tesla scanner and a radial 3D UTE sequence.
Contrast agent was applied intravenously after seven minutes and dynamic contrast enhanced imaging was performed for 21 minutes. The relative enhancement was measured across the imaging time. For quantitive analysis, the enhancement intensity were binned using 6 thresholds; enhancement bins were then compared and statistically analyzed.
The main findings of this study were an increased lung volume between experimental and control group at day 28, determined by manual lung segmentation and a significantly increased DCE during inflammation stage at day 7. Interestingly that appeared not to be any different contrast enhancement in experimental group at day 28 compared to the control group.
1. Please specify the number of used rodents in the study. That included number is not given in the abstract, please add the number of overall included animals here as well as how many were used in experimental and control group. Please also add this in the method section.

*We thank the reviewer for this suggestion, and we have rephrased the abstract regarding controls and experimental numbers in the abstract with the following line: “Eleven male Sprague-Dawley rats received a single intratracheal dose of bleomycin (1000iU) and four rats were included as controls. Imaging was on days 7 and 28 post-installation. “*

I’m not sure if there is data inconsistency here: in the method section you state that n = 15 animals were included. I cannot find any information in the method section how many animals were resorted to the control and to the experimental group.

In figure 1A the n=4 control and n=12 bleomycin rats we’re included which sums in a total of 16 animals. In figure 1B it is not understandable why there are only six animals in the experimental group at a seven and five at day 28.

*We thank the reviewer for bringing this to our attention and we clarified in our text and figures to better describe the group sizes. The group size of 16 animals in the weight curve was an unfortunate error and has been corrected in the revised version to the correct number of 15 animals for the whole group.*

Please revise your numbers throughout the whole manuscript, check how many animals were used and please add this information as I requested above. Whenever there are different numbers at different time points (day 7 and day 28) please explain the missing n here!

*We agree with reviewer nr 2 that the description of the group sizes and which animals underwent MRI examination was lacking and we apologize for this confusion.*

*The following was added to the material and methods to clarify the animal group sizes.*

*A total of 15 animals were included in this study. 4 animals were used as controls (receiving only Saline instillation) and 11 animals received bleomycin. Based on prior experiences with the bleomycin model, 5 out of 11 animals were designated as backup subjects from the imaging group of bleomycin exposed animals, to maintain the study's reliability and continuity in case of unexpected issues with the primary subjects, such as extensive weight loss or imaging or anesthesia related events. Day 7 and day 28 were chosen for DCE-MRI scan days as two representative days of dominant inflammatory and fibrotic time points respectively. The planned group size for DCE-MRI in bleomycin exposed animals was n=6.*

*We also added the following section to the Results section.*

*DCE-MRI was successful in 3 out of 4 control animals on day 7, for 1 animal the Venflon catheter punctured the venous artery and substantial amount of fluid was injected extra-vascular in the tail. This animal was removed from the DCE-MRI on day 28 due to vascular damage in the tail. Subsequently, only 3 controls were available for DCE-MRI on day 28 as well. In the bleomycin-exposed group, all DCE-MRI injections (n=6) were completed successfully on day 7. On day 28, one contrast agent injection partly failed as the images showed that only a fraction of the contrast agent reached the lung. This reduced the group size for DCE-MRI in bleomycin-exposed group animals to n=5.*

2. Regarding the used UTE sequence: please add the radio trajectory information in the abstract as well. The TE of > 0.3 ms appears quite long here, additionally the flip angle is rather high, this is probably why you only get very low signal from the lung tissue. Was this anticipated? Is this a limit from the Bruker sequence or could TE be reduced? Please comment on this. You further state that the flip angle was set to 40° because of sensitivity to Gd contrast agent, was this done empirically or did you perform any estimations?

*The echo time is substantially longer than can be achieved in a 3D-UTE approach as the slice selection takes time in the 2D-UTE version of the sequence. A 3D-UTE version was our preferred approach. Due to hardware limitations, this turned out to be not a viable option (see discussion on this topic in the manuscript). We agree that the flip angle is high with respect to the reported repetition time of 3 ms of the sequence. But as we mention in the material and method section, the way the sequence is written in the Bruker software, means this repetition time is incorrect with multi slice experiments. Due to the looping structure of the sequence code, spokes are acquired for all slices at each spoke angle before acquisition of the next angle is started. The effective repetition time for each slice was therefore much longer than 3 ms, and is depending on the number of slices. The effective true repetition time can be calculated to 114 ms in our situation (38 slices, TR=3ms).*

*We used a visual inspection of the contrast for a single slice with low flip angle compared to the 38 slices with high flip angle. To compensate for the longer repetition time using the 38 slices, the flip angle should preferable have been larger than 40 degrees and the flip angle used is a compromise between T1-weighting, hardware heating and the RF power level used for 28 minutes continuous high duty cycle scanning*

*We have rewritten this section of the manuscript to better clarify this.*

3. In the method section, you explained that images were acquired in coronal orientation. In the figures you show transverse images, why did you do that? Pixel resolution was anisotropic with 0.6 x 0.6 x 1.2 mm and you even zerofill to 0.3 x 0.3? This seems odd, did I miss something here? Please comment or elaborate.

*The respiration triggered gradient echo-based images were acquired in the coronal orientation as an anatomical reference scan for planning the positioning of the 38 slices of the axial/transversal DCE-UTE images. Further, these gradient echo images were used for signal to noise calculations to check the effect of the weight of the animals on coil load and image quality as described. We have emphasized this in the text.*

*The reconstruction and regridding of the radial data are done at a final resolution that is twice the original one in-plane. We must consider that the scans were not triggered to cardiac and respiratory movement. Movement of the lung tissue closer to the diaphragm easily exceeds 5 mm, so any position in the lung has some signal smearing due to movement and is largest in the slice direction. For that reason, no interpolation has been applied in the slice direction.*

4. The binning method that you used for quantification of the DCE signal is very hard to understand! This needs more explanation in the methods section and probably a graphical explanation in a figure, so that its easier to follow.

*The binning method is basically reorganizing the data into a histogram with, in our case, only 7 classes. The difference with a classic histogram is that the classes in our approach have different interval sizes. The complexity of the noisy DCE enhancement data is reduced into 7 bins and allows for looking at average curves and the volume that each interval represents in the lung of the animal.*

*We have rephrased this section in the material and methods and hope that the with the changes implemented in the material and method section, the explanation is sufficient.*

5. Please try and elaborate why there is no difference in DCE in bleomycin day 28 images. I would definitely expect extracellular accumulation of GBCA during washout phase and some kind of late enhancement when there would be fibrosis, but this cannot be seen here. Why?

*The authors thank you for this question. Changes in the dynamics of the DCE enhancement, and most likely changes in the washout period, were one of the assumptions in our study to prove in the bleomycin exposed rats. We can only conclude that based on our data we cannot show that. As explained above, the binning approach didn’t reveal any difference in the dynamics.*

*Analysis close to the major airways is a challenge for the DCE experiment due to the high signal intensity of the vessels and the need to analyze the dynamics in tissue in proximity with these vessels and airways. The vessels pulsate with the heartbeat and the respiration creates substantial movement of tissue, vessels and airways. The data shown is not corrected for either of these physiological processes and signal bleed is to be expected.*

*Further it might be that the echo times used was not short enough to detect the collagen rich areas in the lung, even before administration of the contrast agent. Enhancement with a contrast agent will not help to fix this as T2*-relaxation would be the dominating factor. For this reason, we suggest that future experiments should be carried out at a lower magnetic field strength where many of these susceptibility effects are less of an issue compared to 9.4T.*

*These points have been slightly rewritten in the discussion section to better describe the possible explanations of why substantial differences in DCE dynamics on day 28 could not be observed.*

Please review the manuscript regarding spelling and grammar. If possible, it should be revised by a native speaker. The manuscript contains some very complicated non idiomatic sentence structures and a few spelling errors.

*We have extensively reviewed the manuscript, corrected spelling errors and reformulated sections to improve readability. We like to mention that one of the co-authors is native English speaker.*
